# Subcortical grey matter volume and asymmetry in the long-term course of Rasmussen’s encephalitis

**DOI:** 10.1093/braincomms/fcad324

**Published:** 2023-11-25

**Authors:** Tobias Bauer, Johannes T Reiter, Selma Enders, Vera C W Keil, Alexander Radbruch, Christoph Helmstaedter, Rainer Surges, Theodor Rüber

**Affiliations:** Department of Epileptology, University Hospital Bonn, 53127 Bonn, Germany; Department of Epileptology, University Hospital Bonn, 53127 Bonn, Germany; Department of Epileptology, University Hospital Bonn, 53127 Bonn, Germany; Department of Neuroradiology, University Hospital Bonn, 53127 Bonn, Germany; Department of Radiology and Nuclear Medicine, Amsterdam UMC Location Vrije Universiteit Amsterdam, 1081 HV Amsterdam, Netherlands; Brain Imaging, Amsterdam Neuroscience, Amsterdam UMC, 1081 HV Amsterdam, Netherlands; Cancer Center Amsterdam, Amsterdam UMC, 1081 HV Amsterdam, Netherlands; Department of Neuroradiology, University Hospital Bonn, 53127 Bonn, Germany; Department of Epileptology, University Hospital Bonn, 53127 Bonn, Germany; Department of Epileptology, University Hospital Bonn, 53127 Bonn, Germany; Department of Epileptology, University Hospital Bonn, 53127 Bonn, Germany

**Keywords:** epilepsy, neuroimaging, volumetry, neuroplasticity, longitudinal modelling

## Abstract

Rasmussen’s encephalitis is characterized by drug-resistant focal seizures and chronic inflammation of one hemisphere leading to progressive loss of hemispheric volume. In this cohort study, we aimed to investigate subcortical grey matter volumes and asymmetries in Rasmussen’s encephalitis longitudinally in clinically relevant subgroups. We retrospectively included all T_1_-weighted MRI scans of all people with Rasmussen’s encephalitis who were treated at the University Hospital Bonn between 1995 and 2022 (*n* = 56, 345 scans, median onset 8 years, 36 female). All cases were classified as type 1 (onset ≤ 6 years) or type 2 (onset > 6 years). Subcortical segmentations were performed using FreeSurfer. Longitudinal trajectories of subcortical volumes and hemispheric ratios (ipsi-/contralesional) were assessed using linear mixed-effect models. Unihemispheric cortical degeneration was accompanied by ipsilesional atrophy of the nucleus accumbens, caudate nucleus, putamen, thalamus and contralesional atrophy of the nucleus accumbens and caudate nucleus both in type 1 (all *P* ≤ 0.014) and type 2 (all *P* < 0.001). In type 1, however, contralesional volume increase of the amygdala, hippocampus, pallidum and thalamus was found (all *P* ≤ 0.013). Both ipsilesional and contralesional subcortical atrophies, like cortical atrophy, are most probably caused by neurodegeneration following chronic neuroinflammation. We speculate that contralesional volume increase in type 1 could be related to either neuroplasticity or ongoing acute neuroinflammation, which needs to be investigated in further studies.

## Teaser

Bauer *et al*. investigate subcortical grey matter in Rasmussen’s encephalitis longitudinally. They found ipsilesional (nucleus accumbens, caudate nucleus, putamen, thalamus) and contralesional (nucleus accumbens, caudate nucleus) atrophy. In type 1, but not 2, they observed contralesional volume increase (amygdala, hippocampus, pallidum, thalamus), which may be related to neuroplasticity or neuroinflammation.

## Graphical Abstract

**Graphical Abstract:**
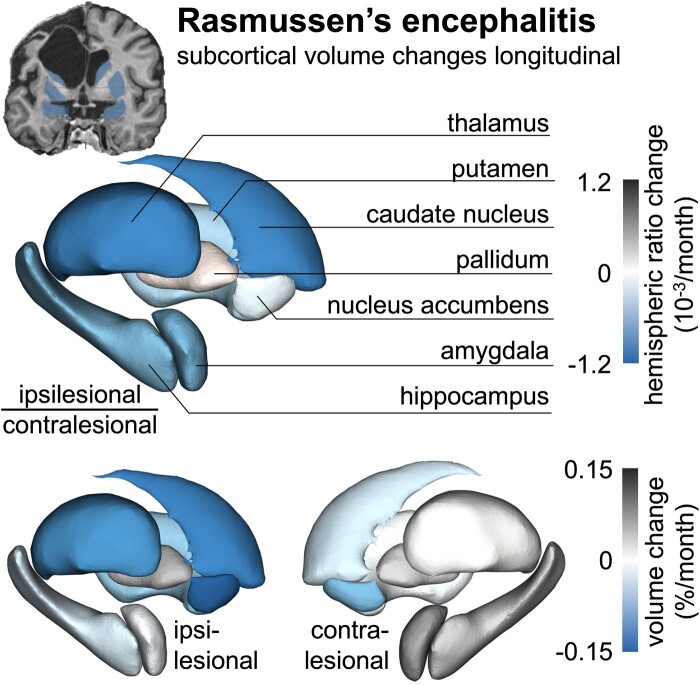

## Introduction

Rasmussen’s encephalitis (RE) is a rare neurological disorder that is characterized by drug-resistant focal seizures and chronic inflammation of one hemisphere leading to progressive loss of hemispheric volume and cognitive function.^1^ Since its first mention in literature,^2^ two clinical characteristics are consistently named: first, its strictly unihemispheric manifestation. Second, RE affects primarily children and young adults, with the median age of disease onset usually reported around the age of 6 years.^3-5^ However, there are case reports and smaller case series questioning these two characteristics by describing atypical courses of RE, including bilateral presentations and late-onset RE.^6-11^ This is reflected in a classification proposed by Bien and colleagues,^3^ who distinguish between typical cases with short prodromal phase (< 8 months) and disease onset earlier than 6 years of age, to which they refer as ‘type 1’, and atypical cases with longer prodromal phase or later disease onset, referred to as ‘type 2’.

To quantify the unihemispheric cortical degeneration over the years of disease progress, the MRI-based hemispheric ratio (HR) is considered a reliable imaging biomarker to monitor disease progression.^3,12-14^ However, it has been suggested that the disease process of RE is not limited to the neocortex of the lesional hemisphere: previous studies applying volumetric image analyses in RE have found both lower and higher neocortical volumes in the contralesional hemisphere relative to control subjects.^12,15,16^ Furthermore, it has been shown that in addition to the neocortex, cerebellar degeneration may occur.^17^ Subcortical grey matter, especially the basal ganglia, have been repeatedly described to degenerate along with cortical grey matter,^18-20^ and the ipsilateral atrophy of the head of the caudate nucleus is considered to be an early diagnostic sign.^21^ However, volumetric trajectories of subcortical grey matter have not yet been systematically investigated in RE. While the basal ganglia are critical for the regulation of movement, sensation and cognition and are affected in a variety of neurological diseases,^22^ the hippocampus and amygdala are central structures in many epileptic and neurodegenerative disorders.^23-25^

It is the aim of this study is to investigate different longitudinal patterns of volumetric changes and asymmetries of subcortical grey matter structures in clinically relevant subgroups of RE and to relate these potential new imaging markers to cortical HR as a well-established imaging marker in RE.

## Materials and methods

### Participants

We retrospectively ascertained all people with RE who were treated at the Department of Epileptology of the University Hospital Bonn between 1995 and 2022 and had at least one T_1_-weighted MRI available that was acquired within 20 years (240 months) after disease onset. In all cases, diagnosis of RE was made according to diagnostic criteria set forth by Bien and colleagues,^14^ including the addition by Olson and colleagues.^26^ We classified all subjects as type 1 (typical RE, age at onset ≤6 years and duration of prodromal stage ≤8 months) or type 2 (atypical/late-onset RE, age at onset > 6 years or duration of prodromal stage >8 months) as proposed by Bien and colleagues.^3^ The study was approved by the Institutional Review Board of the University Hospital Bonn, and all subjects or their legal guardians provided written informed consent.

### MRI acquisition and analysis

Isotropic T_1_-weighted MRI scans (all voxel size ≤ 1 mm) were acquired on 1.5 T or 3 T scanners at the University Hospital Bonn either at the Department of Neuroradiology or at the Life and Brain Centre. Scan parameters differ between scanners and are detailed in Supplementary Table 1.

Segmentation and cortical reconstruction of T1-weighted MRI scans was performed using the longitudinal framework in FreeSurfer (v6.0, https://surfer.nmr.mgh.harvard.edu/). To account for the respective ipsilesional and contralesional hemispheres, images of those individuals with a right hemispheric disease focus were flipped along the *x*-axis and the symmetric ‘fsaverage_sym’ template was used with the FreeSurfer pipeline. Volumes of seven subcortical structures (nucleus accumbens, amygdala, caudate nucleus, hippocampus, pallidum, putamen and thalamus) and of the cortex were extracted from the FreeSurfer output and expressed as proportion of the estimated total intracranial volume (eTIV) provided by FreeSurfer. The cortical HR was calculated as the ipsilesional/contralesional ratio of the cortical volume. For all analysed subcortical structures, a subcortical HR was calculated analogously.

### Statistical analysis

We performed all statistical analyses using the ‘SciPy’ and ‘statsmodels’ module in ‘Python’. In mixed-effects linear models, which we used to be able to handle any number of MRI scans per subject, volumetric data or HR was set as dependent variables. In the first model, ‘age at disease onset’, **‘**sex’, ‘lesional hemisphere’ and ‘disease duration’ were included as fixed effects. In the second model, the interaction ‘disease duration’ by ‘subgroup’ (type 1 RE or type 2 RE) was added as fixed effect. In the third model, ‘age at disease onset’, **‘**sex’, ‘lesional hemisphere’ and ‘cortical HR’ were set as fixed effects. A subject-level intercept was included as a random effect in all models. To compare continuous data between subgroups, Mann–Whitney U-test was used. Categorical data between subgroups were compared using Fisher’s exact test. We consider an effect to be significant if *P* < 0.05.

## Results

### Participants

We included 56 individuals with RE (36 female, median age at onset 8 years, range 1–51), with a total of 345 T_1_-weighted MRI scans (median 6 per case, range 1–17). Data from parts of this cohort have previously been published by Wagner and colleagues,^13^ David, Prillwitz and colleagues,^15^ and Reiter and colleagues.^17^ When comparing type 1 RE and type 2 RE, contralesional abnormalities in EEG (ictal onset or interictal epileptiform discharges) were significantly more frequent in type 1 RE than in type 2 RE (*P* = 0.003). There was no significant group difference regarding sex, lesional hemisphere, number of MRI scans, disease duration at MRI and the use of immunotherapy between type 1 RE and type 2 RE. All group characteristics are summarized in Table 1.

**Table 1:** Summary of group characteristics

Variable	All	Type 1	Type 2	*P*-value
*n* cases	56	26	30	n/a
Female, *n* (%)	36 (64)	15 (58)	21 (70)	0.41
Total MRI scans	345	151	194	n/a
MRI scans per case, median (range)	6 (1–17)	5.5 (1–15)	6 (1–17)	0.70
Age at onset (years), median (range)	8 (1–51)	5 (1–6)	14.5 (7–51)	**<0.001**
Age at first MRI (years), median (range)	13 (2–57)	6 (2–48)	22.5 (8–57)	**<0.001**
Duration at first MRI (months), median (range)	25 (0–196)	15.5 (0–196)	39.5 (0–211)	0.10
Age at last MRI (years), median (range)	17 (2–61)	10 (2–48)	27.5 (8–61)	**<0.001**
Duration at last MRI (months), median (range)	94 (2–240)	74 (2–240)	119.5 (2–240)	0.23
Median duration at MRI (months), median (range)	54.5 (1.5–211)	37 (1.5–206)	55 (8–211)	0.15
Left lesional, *n* (%)	32 (57)	14 (54)	18 (60)	0.78
Contralesional EEG, *n* (%)	24 (43)	17 (65)	7 (23)	**0**.**003**
Immunotherapy, *n* (%)	46 (82)	20 (77)	26 (87)	0.49
Hemispherotomy, *n* (%)	14 (25)	12 (46)	2 (7)	**0**.**002**

Statistical tests were performed to compare type 1 and type 2. *P*-values refer to Mann–Whitney U-test for continuous data or Fisher’s exact test for categorical data. Significant (*P* < 0.05) test results are printed in bold.

### Subcortical HR and subcortical volumes

**Figure 1:**
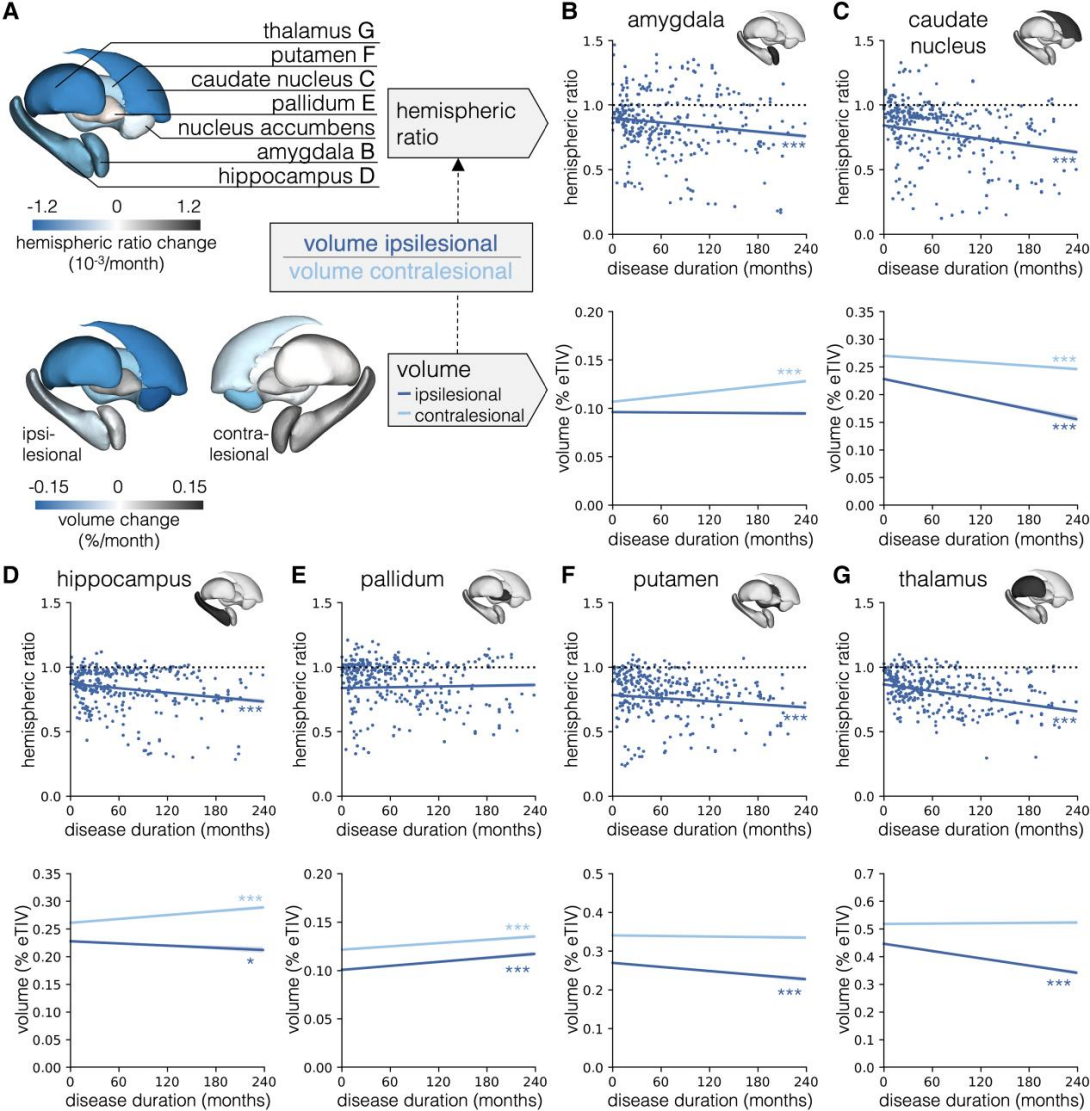
**Graphical representation of longitudinal mixed-effects models.** (**A**) Visualization of regression slopes for all seven subcortical structures on a 3D model; HRs in the top panel and volumes in the lower panel. Regression lines with 95% confidence bands for the (**B**) amygdala, (**C**) caudate nucleus, (**D**) hippocampus, (**E**) pallidum, (**F**) putamen, and (**G**) thalamus; HRs in top panels and volumes as proportion of eTIV in bottom panels. A regression plot for the nucleus accumbens is not shown. eTIV, estimated total intracranial volume; *P < 0.05, ***P < 0.001.

Across all cases, we found a significant decrease of the subcortical HR of all structures (all *P* < 0.001), except for the pallidum. We observed a significant decrease of the ipsilesional volume of the nucleus accumbens (*P* < 0.001), caudate nucleus (*P* < 0.001), hippocampus (*P* = 0.04), putamen (*P* < 0.001) and thalamus (*P* < 0.001) and a significant volume increase of the ipsilesional pallidum (*P* < 0.001). In the contralesional hemisphere, we found a significant volume decrease of the nucleus accumbens and caudate nucleus (both *P* < 0.001), as well as a significant volume increase of the amygdala, hippocampus and pallidum (all *P* < 0.001). Statistical details of the mixed-effects models are presented in Supplementary Table 2 and visualized in Fig. 1.

### Subcortical HR and subcortical volumes in type 1 RE versus type 2 RE

**Figure 2:**
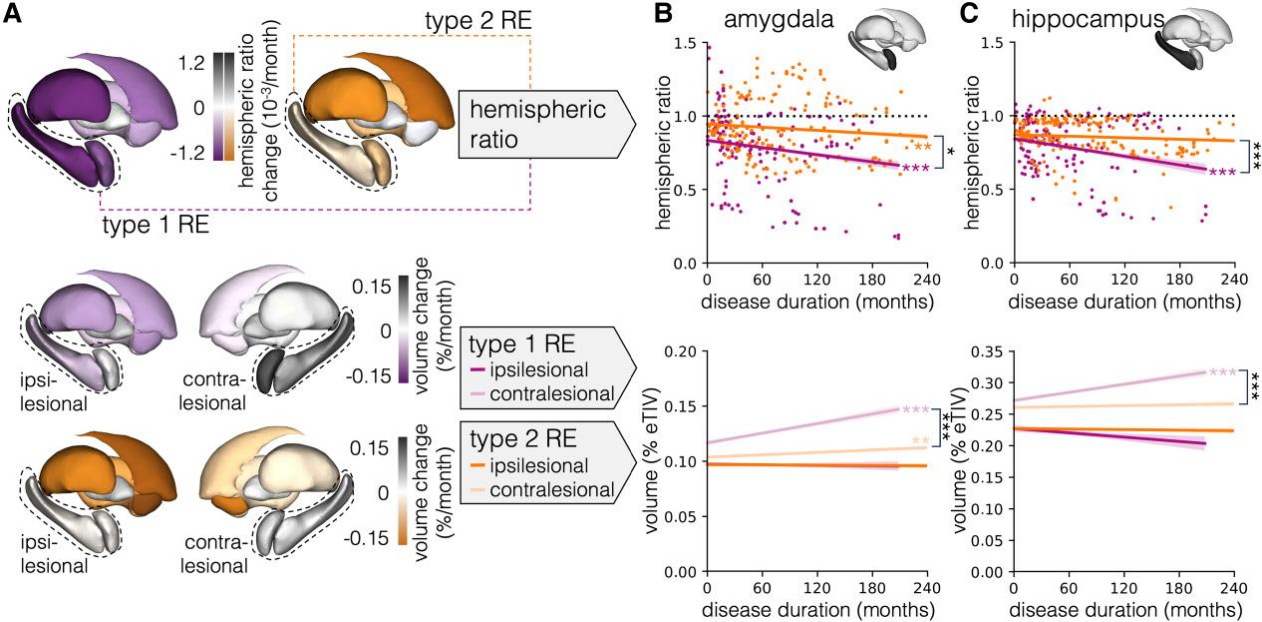
**Graphical representation of mixed-effects models comparing type 1 and type 2 RE.** (**A**) Visualization of regression slopes for all seven subcortical structures on a 3D model; HRs for type 1 RE and type 2 RE in the top panel and type 1 RE and type 2 RE volumes in lower panels. Regression lines with 95% confidence bands for the (**B**) amygdala and (**C**) hippocampus; HRs in top panels and volumes as proportion of eTIV in bottom panels. Only regression plots for structures with significant HR differences between type 1 RE and type 2 RE are shown. eTIV, estimated total intracranial volume; RE, Rasmussen’s encephalitis, *P < 0.05, **P < 0.01, ***P < 0.001.

When comparing both types, we found a significantly stronger decrease of the subcortical HR of the amygdala (*P* = 0.034) and hippocampus (*P* < 0.001) driven by a significantly stronger increase of the contralesional volume (both *P* < 0.001) in type 1 RE than in type 2. Furthermore, we observed a significantly stronger increase of the contralesional volume of the pallidum (*P* = 0.013), putamen (*P* = 0.017) and thalamus (*P* < 0.001) in type 1 RE than in type 2. Results of all mixed-effects models comparing HR and volumetric changes in type 1 RE and type 2 RE are listed in Supplementary Table 3 and shown in Fig. 2.

### Correlation between subcortical and cortical HR in type 1 versus type 2 RE

**Figure 3:**
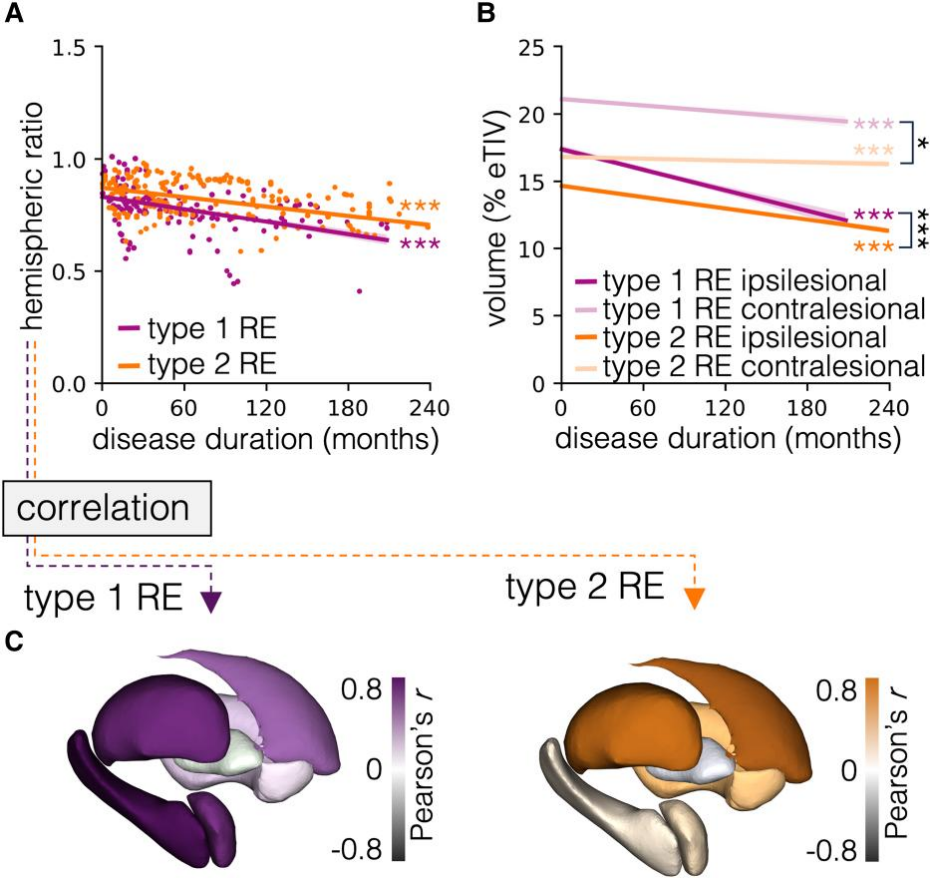
**Graphical representation of mixed-effects models assessing cortical HRs longitudinally and its associations with subcortical HRs.** Regression lines with 95% confidence bands for cortical (**A**) HRs and (**B**) volumes as proportion of eTIV. (**C**) Visualization of Pearson’s correlation coefficients between cortical and subcortical HRs for all seven subcortical structures on a 3D model in type 1 RE (left) and type 2 RE (right). eTIV, estimated total intracranial volume; RE, Rasmussen’s encephalitis, *P < 0.05, ***P < 0.001.

The cortical HR significantly decreased in type 1 RE and type 2 RE (both *P* < 0.001). In mixed-effects models assessing associations between the cortical and subcortical HR, we found a significant association between the cortical HR and the HR of amygdala, caudate nucleus, hippocampus, putamen and thalamus (all *P* < 0.001) in type 1 RE and between the cortical HR and the HR of the caudate nucleus, putamen and thalamus (all *P* < 0.001) in type 2 RE. When comparing both types, we found a significantly stronger association between the cortical HR and the HR of the amygdala and hippocampus (both *P* < 0.001) in type 1 RE than in type 2 RE. Results of mixed-effects models exploring associations between the cortical and subcortical HRs are displayed in Supplementary Table 4 and visualized in Fig. 3.

## Discussion

We performed segmentation of 7 subcortical grey matter structures and of the cortex in 345 MRI scans along the disease process of 56 individuals with RE. Subcortical structures showed ipsilesional volume decrease, except for the pallidum. Contralesional volumes showed either volume decrease (nucleus accumbens, caudate nucleus) or volume increase (amygdala, hippocampus, pallidum). When comparing type 1 RE and type 2 RE, the contralesional volume increase of mesiotemporal structures and the thalamus is only seen in type 1 RE. The association between cortical HR and HR of the mesiotemporal structures is also specific for type 1 RE.

A decrease of the HR of the caudate nucleus, putamen and thalamus along the disease process was found independent of type 1 RE and type 2 RE, and their HR was further correlated with the cortical HR. In type 2 RE, the HR decrease is caused by volume decrease in the ipsilesional hemisphere together with a less pronounced decrease in the contralesional side. In type 1 RE, in contrast, the HR decrease of the thalamus and putamen is a result of volume decrease in the ipsilesional hemisphere and volume increase in the contralesional side. This illustrates that it can be misleading to only look at HR in this scenario.

Hyperintense signal change or atrophy of the head of the ipsilesional caudate nucleus may be an early sign for RE;^14^ it is therefore not surprising that atrophy continues along the disease process. However, the observation that atrophy of the caudate nucleus also occurs in the contralesional side is new. Imaging abnormalities of the ipsilesional putamen have also been reported before,^18-21^ but ipsilesional thalamic atrophy is a novel finding in RE.

In type 2 RE, mesiotemporal volumes were lower in the ipsilesional than in the contralesional hemisphere, and HR and volumes remained stable along the disease process. In type 1 RE, in contrast, we observed ipsilesional volume decrease and contralesional volume increase, which leads to a decrease in the respective HR. Furthermore, the HRs of the hippocampus and amygdala were correlated with cortical HR in type 1 RE, but not in type 2 RE. With this pattern of mesiotemporal involvement, type 1 RE and type 2 RE can be specifically distinguished on a group level. This underlines the value of subcortical HR as a potential imaging biomarker in the diagnosis of RE, if further validated on a single-case level in future studies.

Ipsilesional atrophy of subcortical grey matter is most likely caused by neuronal cell loss, in analogy to ipsilesional cortical atrophy and as a direct consequence of the primary disease process of RE. For the increase of several contralesional subcortical volumes, which has also been described to occur in the contralesional cortex,^15^ there are two possible explanations. At first, contralesional volume increase may fall under the general realm of neuroplasticity. Contralesional hippocampal volume increase was also found after childhood arterial ischaemic stroke in younger children aged younger than 9 years at stroke, while this was not observed in children aged older than 9 years.^27^ Despite different age cut-offs between age groups, this is in line with our finding of contralesional hippocampal volume increase in type 1 RE, but not type 2 RE. A second explanation could be possible inflammatory processes in the contralateral hemisphere. At the onset of the disease, RE may show hyperintensity in the ipsilesional grey matter, which only later transitions to atrophy. This sequence of signal increase with accompanying volume increase in the acute stage, followed by atrophy in the chronic stage, is thought to be caused by a cytotoxic oedema, which also occurs in status epilepticus or autoimmune encephalitis.^28,29^ This aligns with our observation that volume increase of contralesional mesiotemporal structures is found only in type 1 RE, since in these cases contralesional epileptiform EEG findings were more frequent than in type 2 RE. This hints towards ongoing contralesional inflammation and fits in the picture that the contralesional hemisphere in RE is not unaffected, at least when contralesional epileptiform EEG patterns are present.^15^ Whereas the analogy to trajectories of subcortical volumes after arterial ischaemic stroke is more suggestive of neuroplasticity, the association with autoimmune encephalitis points to neuroinflammation. However, both explanations remain speculative, and the underlying processes could as well be a combination of both.

Lastly, it must be considered that the MRI scans included in this study cover a wide age range (2–61 years), encompassing infantile, juvenile and adult brains. While attempts were made to best address the morphometric differences by including the age at onset as independent variable in the statistical models and using volumes relative to the eTIV, it should be noted that neither approach fully represents the profound differences between developing childhood and fully mature adult brains.

### Strengths and limitations

The main strength of our study lies in the large retrospective data set paired with powerful statistical modelling. Linear mixed-effects models, such as those used in this study, allow modelling of long-term longitudinal trajectories with an arbitrary number of MRI time points per subject without the need to define discrete disease stages beforehand, making them ideal for this scenario.

However, these strengths also result in three limitations. First, like every retrospective investigation, our study is challenged by therapeutic interventions, including anti-seizure medication and immunomodulatory treatment. Any large cohort, such as the one analysed in this study, is inherently too heterogeneous in this regard to allow for these effects to be controlled in statistical models. Second, MRI scans were performed with different technical setups depending on clinical needs, which is another confounding effect. Third, while the mixed-effects regression model is suitable to consider individual disease courses through random effects, all effects are estimated in a linear way and, thus, ignore cyclic or nonlinear trajectories.

## Conclusion

Our retrospective longitudinal study demonstrates that the unihemispheric cortical degeneration characteristic of RE is paralleled by ipsilesional atrophy of the basal ganglia and thalamus. In addition, mesiotemporal involvement with contralesional volume increase is found only in type 1 RE, but not in type 2 RE. Overall, our results imply that subcortical structures should be given more attention in the diagnosis and disease monitoring of RE and that subcortical volumes should be further validated as additional imaging biomarkers besides conventional HR.

## Supplementary Material

fcad324_Supplementary_Data

## Data Availability

The data that support the findings of this study are available on request from the corresponding author. The data are not publicly available due to privacy or ethical restrictions.
