# Supplementary material for: Subcortical grey matter volume and asymmetry in the long-term course of Rasmussen’s encephalitis
Source: Brain Commun. 2023 Nov 25;5(6):fcad324. doi: 10.1093/braincomms/fcad324 (PMC10710296; doi:10.1093/braincomms/fcad324)
Supplement: fcad324_Supplementary_Data [file fcad324_supplementary_data.pdf]

Supplementary Table 1. MRI sequences.
Sequence parameters of all T1-weighted MRI scans included in this study. MPRAGE: magnetization prepared rapid gradient echo, TFE: turbo field echo.

site	n scans	model	B0 (T)	acquisition type	TR (ms)	TE (ms)	flip angle (°)	voxel size (mm)
Department of Neuroradiology	77	Philips Intera	1.5	TFE	15.26	3.60	30	1.0
	106	Philips Intera	3	TFE	8.26	3.74	8	1.0
	54	Philips Achieva	3	TFE	8.11	3.78	8	1.0
Life and Brain Center	2	Siemens Avanto	1.5	MPRAGE	1660	3.93	15	1.0
	72	Siemens Trio	3	MPRAGE	1570	3.42	15	1.0
	34	Siemens TrioTim	3	MPRAGE	1660	2.54	9	0.8

Supplementary Table 2. Results of all mixed-effects models.
P-values refer to *t*-tests of the parameter estimates versus 0. Significant ($P < 0.05$) test results are printed in bold. 2.5%/97.5%-CL: 2.5%/97.5% confidence limit of parameter estimate, HR: hemispheric ratio.

structure	measure	estimate	2.5%-CL	97.5%-CL	*P*-value
nucleus accumbens	HR ($x10^{-3}$/month)	-0.154	-0.894	0.587	0.684
	ipsilesional volume (cm^3/month)	-0.042	-0.056	-0.028	**< 0.001**
	contralesional volume (cm^3/month)	-0.023	-0.036	-0.009	**0.001**
amygdala	HR ($x10^{-3}$/month)	-0.596	-0.804	-0.388	**< 0.001**
	ipsilesional volume (cm^3/month)	-0.006	-0.032	0.019	0.633
	contralesional volume (cm^3/month)	0.088	0.064	0.112	**< 0.001**
caudate nucleus	HR ($x10^{-3}$/month)	-0.867	-1.079	-0.655	**< 0.001**
	ipsilesional volume (cm^3/month)	-0.304	-0.368	-0.240	**< 0.001**
	contralesional volume (cm^3/month)	-0.101	-0.138	-0.065	**< 0.001**
hippocampus	HR ($x10^{-3}$/month)	-0.569	-0.779	-0.358	**< 0.001**
	ipsilesional volume (cm^3/month)	-0.066	-0.128	-0.003	**0.039**
	contralesional volume (cm^3/month)	0.117	0.072	0.162	**< 0.001**
pallidum	HR ($x10^{-3}$/month)	0.104	-0.099	0.307	0.315
	ipsilesional volume (cm^3/month)	0.069	0.042	0.095	**< 0.001**
	contralesional volume (cm^3/month)	0.058	0.034	0.081	**< 0.001**
putamen	HR ($x10^{-3}$/month)	-0.401	-0.568	-0.235	**< 0.001**
	ipsilesional volume (cm^3/month)	-0.176	-0.241	-0.110	**< 0.001**
	contralesional volume (cm^3/month)	-0.025	-0.082	0.032	0.396
thalamus	HR ($x10^{-3}$/month)	-0.874	-1.036	-0.713	**< 0.001**
	ipsilesional volume (cm^3/month)	-0.439	-0.521	-0.358	**< 0.001**
	contralesional volume (cm^3/month)	0.022	-0.056	0.100	0.581

Supplementary Table 3. Results of all mixed-effects models comparing type 1 and 2. *P*-values refer to *t*-tests of the parameter estimates versus 0, and type 1 RE versus type 2 RE. Significant ($P < 0.05$) test results are printed in bold. 2.5%/97.5%-CL: 2.5%/97.5% confidence limit of parameter estimate, HR: hemispheric ratio.

structure	measure	RE type	estimate	2.5%-CL	97.5%-CL	*P*-value (vs. 0)	*P*-value (type 1 vs. 2 RE)
nucleus accumbens	HR ($x10^{-3}$/month)	1	-0.522	-1.192	0.149	0.127	0.361
		2	0.130	-1.097	1.357	0.835	
	ipsilesional volume (cm^3/month)	1	-0.038	-0.060	-0.016	**0.001**	0.775
		2	-0.042	-0.060	-0.024	**< 0.001**	
	contralesional volume (cm^3/month)	1	-0.009	-0.026	0.007	0.270	0.066
		2	-0.032	-0.050	-0.014	**0.001**	
amygdala	HR ($x10^{-3}$/month)	1	-0.815	-1.151	-0.479	**< 0.001**	**0.034**
		2	-0.359	-0.613	-0.105	**0.006**	
	ipsilesional volume (cm^3/month)	1	-0.007	-0.051	0.037	0.755	0.948
		2	-0.005	-0.034	0.023	0.717	
	contralesional volume (cm^3/month)	1	0.145	0.101	0.189	**< 0.001**	**< 0.001**
		2	0.036	0.014	0.057	**0.001**	
caudate nucleus	HR ($x10^{-3}$/month)	1	-0.786	-1.084	-0.488	**< 0.001**	0.502
		2	-0.931	-1.233	-0.630	**< 0.001**	
	ipsilesional volume (cm^3/month)	1	-0.287	-0.381	-0.193	**< 0.001**	0.659
		2	-0.316	-0.403	-0.229	**< 0.001**	
	contralesional volume (cm^3/month)	1	-0.084	-0.136	-0.033	**0.001**	0.410
		2	-0.115	-0.166	-0.063	**< 0.001**	
hippocampus	HR ($x10^{-3}$/month)	1	-0.989	-1.390	-0.587	**< 0.001**	**< 0.001**
		2	-0.167	-0.341	0.007	0.060	
	ipsilesional volume (cm^3/month)	1	-0.114	-0.232	0.004	0.059	0.135
		2	-0.014	-0.071	0.043	0.628	
	contralesional volume (cm^3/month)	1	0.215	0.145	0.285	**< 0.001**	**< 0.001**
		2	0.023	-0.031	0.077	0.401	
pallidum	HR ($x10^{-3}$/month)	1	0.063	-0.236	0.363	0.679	0.687
		2	0.148	-0.130	0.425	0.298	
	ipsilesional volume (cm^3/month)	1	0.087	0.044	0.131	**< 0.001**	0.179
		2	0.051	0.019	0.082	**0.002**	
	contralesional volume (cm^3/month)	1	0.088	0.054	0.122	**< 0.001**	**0.013**
		2	0.029	-0.003	0.061	0.073	
putamen	HR ($x10^{-3}$/month)	1	-0.403	-0.678	-0.128	**0.004**	0.978
		2	-0.399	-0.601	-0.196	**< 0.001**	
	ipsilesional volume (cm^3/month)	1	-0.144	-0.260	-0.029	**0.014**	0.405
		2	-0.202	-0.273	-0.131	**< 0.001**	
	contralesional volume (cm^3/month)	1	0.047	-0.046	0.141	0.322	**0.017**
		2	-0.093	-0.160	-0.026	**0.007**	
thalamus	HR ($x10^{-3}$/month)	1	-1.032	-1.286	-0.778	**< 0.001**	0.066
		2	-0.725	-0.931	-0.519	**< 0.001**	
	ipsilesional volume (cm^3/month)	1	-0.406	-0.529	-0.283	**< 0.001**	0.503
		2	-0.462	-0.570	-0.353	**< 0.001**	
	contralesional volume (cm^3/month)	1	0.164	0.034	0.294	**0.013**	**0.001**
		2	-0.108	-0.197	-0.020	**0.016**	

Supplementary Table 4. Results of all mixed-effects models assessing associations between cortical and subcortical HR

P-values refer to *t*-tests of the parameter estimates versus 0 and type 1 RE versus type 2 RE. Significant (P < 0.05) test results are printed in bold. 2.5%/97.5%-CL: 2.5%/97.5% confidence limit of parameter estimate, HR: hemispheric ratio.

**measure**	**RE type**	**estimate**	**2.5%-CL**	**97.5%-CL**	***P*-value (vs. 0)**	***P*-value (type 1 vs. 2 RE)**
cortical HR ($x10^{-3}$/month)	1	-0.930	-1.171	-0.689	**<0.001**	0.109
	2	-0.696	-0.850	-0.541	**<0.001**	
ipsilesional cortical volume (cm^3/month)	1	-25.306	-30.925	-19.687	**<0.001**	**<0.001**
	2	-14.037	-16.977	-11.096	**<0.001**	
contralesional cortical volume (cm^3/month)	1	-8.004	-12.276	-3.733	**<0.001**	**0.036**
	2	-2.207	-5.540	1.126	**<0.001**	
correlation cortical ~ nucleus accumbens HR	1	0.159	-0.244	0.563	0.439	0.729
	2	0.343	-0.617	1.304	0.483	
correlation cortical ~ amygdala HR	1	0.844	0.683	1.004	**<0.001**	**<0.001**
	2	0.181	-0.024	0.385	0.083	
correlation cortical ~ caudate nucleus HR	1	0.685	0.529	0.841	**<0.001**	0.163
	2	0.883	0.653	1.113	**<0.001**	
correlation cortical ~ hippocampus HR	1	1.097	0.922	1.271	**<0.001**	**<0.001**
	2	0.111	-0.030	0.251	0.122	
correlation cortical ~ pallidum HR	1	-0.099	-0.280	0.083	0.287	0.792
	2	-0.138	-0.362	0.087	0.230	
correlation cortical ~ putamen HR	1	0.308	0.143	0.473	**<0.001**	0.547
	2	0.379	0.217	0.541	**<0.001**	
correlation cortical ~ thalamus HR	1	0.884	0.781	0.987	**<0.001**	0.303
	2	0.789	0.638	0.939	**<0.001**	
